# Renal Artery Vasodilation May Be An Indicator of Successful Sympathetic Nerve Damage During Renal Denervation Procedure

**DOI:** 10.1038/srep37218

**Published:** 2016-11-16

**Authors:** Weijie Chen, Huaan Du, Jiayi Lu, Zhiyu Ling, Yi Long, Yanping Xu, Peilin Xiao, Laxman Gyawali, Kamsang Woo, Yuehui Yin, Bernhard Zrenner

**Affiliations:** 1 Department of Cardiology, the Second Affiliated Hospital of Chongqing Medical University, Chongqing Cardiac Arrhythmias Therapeutic Service Center, Chongqing, China; 2 Department of Cardiolgy, Chongqing Province Hospital of Traditional Chinese Medicine, Chongqing, China; 3 School of Life Sciences, the Chinese University of Hong Kong, Hong Kong, China; 4 Medizinische Klinik I, Krankenhaus Landshut-Achdorf, Landshut, Germany

## Abstract

Autonomic nervous system plays a crucial role in maintaining and regulating vessel tension. Renal denervation (RDN) may induce renal artery vasodilation by damaging renal sympathetic fibers. We conducted this animal study to evaluate whether renal artery vasodilation could be a direct indicator of successful RDN. Twenty-eight Chinese Kunming dogs were randomly assigned into three groups and underwent RDN utilizing temperature-controlled catheter (group A, n = 11) or saline-irrigated catheter (group B, n = 11) or sham procedure (group C, n = 6). Renal angiography, blood pressure (BP) and renal artery vasodilation measurements were performed at baseline, 30-minute, 1-month, and 3-month after interventions. Plasma norepinephrine concentrations were tested at baseline and 3-month after intervention. Results showed that, in addition to significant BP reduction, RDN induced significant renal artery vasodilation. Correlation analyses showed that the induced renal artery vasodilation positively correlated with SBP reduction and plasma norepinephrine reduction over 3 months after ablation. Post hoc analyses showed that saline-irrigated catheter was superior to TC catheter in renal artery vasodilation, especially for the acute dilatation of renal artery at 30-minute after RDN. In conclusion, renal artery vasodilation, induced by RDN, may be a possible indicator of successful renal nerve damage and a predictor of blood pressure response to RDN.

Hypertension is highly prevalent and a proportion of patients have few options once maximally tolerated medical therapy has been exhausted^1^. It has been generally recognized that elevated sympathetic nerve activity, especially the increased sympathetic outflow to the kidneys, plays a pivotal role in development and perpetuation of hypertension, as well as its adverse cardiovascular events^2^,^3^. In line with this notion, catheter based renal denervation (RDN), which targeted at disrupting renal sympathetic nerves, emerged as a logical therapeutic approach for the treatment of hypertension^4^,^5^,^6^.

A series of clinical studies^7^,^8^,^9^,^10^,^11^ indicated that RDN could lead to significant blood pressure (BP) reduction in patients with resistant hypertension until to the Simplicity HTN-3^12^ failed to demonstrate the superiority of RDN over pharmacological management of hypertension no matter for office BP or 24-hour ambulatory BP measurements. However, further evaluation of the Simplicity HTN-3 trial identified several potential confounding factors that may contribute to the negative results, including over-proportion of Afro-American participants, short of operative experience for RDN, insufficient 4-quadrant ablations, and antihypertensive medication changes^13^,^14^. Afterward, the Global SYMPLICITY Registry re-showed significant reduction of office and ambulatory systolic BPs at 6 months after RDN, especially in the cohort with severe hypertension (office systolic BP, ≥160 mm Hg; 24-hour systolic BP, ≥135 mm Hg; and ≥3 antihypertensive medication classes)^15^. Therefore, RDN technique should not be abandoned just because of the negative results of Symplicity HTN-3^16^. In contrast, more questions than definite answers remain after the publication of Symplicity HTN-3. Lack of indicator or predictor of successful renal sympathetic nerve damage on spot was one of the problems in focus.

After the first identifiable description of the anatomy of sympathetic nervous system by Thomas Willis, Pourfour du Petit clarified the neural control of blood vessel calibre and demonstrated conjunctival vessel dilatation after section of cervical sympathetic nerves^17^,^18^. Stelling in 1840 suggested that the vasomotor fibers were sympathetic nerves originating in the central nervous system and supplying the peripheral blood vessels. In 1852, Claude Bernard and others observed dilatation of blood vessels by sectioning sympathetic nerves^18^,^19^. Renal sympathetic fibers enter the hilus of the kidney in association with the renal artery and subsequently terminate on several intra renal elements including renal arterial vascular segments, juxta-glomerular apparatus, and renal tubular cells^20^,^21^,^22^,^23^. On the basis of the above mentioned, we hypothesize that once renal sympathetic fibers are damaged or sufficiently injured by catheter based RDN, in addition to BP reduction, renal artery vasodilation would be observed immediately.

According to our previous experience from the on-going SWAN-HT Study (identifier: NCT01417221), renal artery vasodilation could be observed after RDN using the saline-irrigated catheter (Thermocool^®^ catheter). However, Templin showed that local tissue damage significantly decreased mean diameter of renal artery after RDN performed with the temperature-controlled (TC) radiofrequency (RF) EnligHTN catheter or the Symplicity catheter^24^. Therefore, the aim of this study was to investigate the impact of RDN on renal artery vasodilation and BP reduction via saline-irrigated and temperature-controlled radiofrequency catheters.

## Methods

### Study Subjects

This study was performed on 30 healthy adult Chinese Kunming dogs aging 3–4 years, weighing 30–35 kg. Eligible dogs were older than three years and had systolic BP of 140 mmHg or more. The Chinese Kunming dogs are larger, more aggressive and fierce than beagles, spaniels and mongrels, more importantly having high natural BP and sympathetic activity^25^,^26^, which are ideal as the experimental animals for renal denervation studies. Using the Chinese Kunming dogs as the experimental animals, we had successfully performed a series of basic researches related to catheter-based RDN technique^25^,^27^,^28^. Prior to conducting experiments in Chinese Kunming dogs, the reasons related to using Chinese Kunming dogs as experimental animals, the source, breeding, feeding conditions, and the experimental protocol of this study was reviewed and approved by the animal experimentation ethics committee of the Chongqing Medical University, following the guidelines of the National Institutes of Health and of the Declaration of Helsinki for the care and use of laboratory animals. All enrolled dogs of this study were obtained from Chengdu Breeding Base of Chinese Kunming Dogs in Sichuan province in China, and standardly fed in Chongqing Medical University Laboratory Animal Center throughout the experimental period.

### Experiment Protocol and Group Setting

The dogs were randomly assigned in a 2:2:1 ratio to undergo RDN utilizing TC catheter (group A, n = 12) or RDN utilizing saline-irrigated catheter (group B, n = 12) or a sham procedure in control group (group C, n = 6). Renal angiography and invasive BP measurement via femoral artery were performed under anesthesia at baseline, 30 minutes, 1 month, and 3 months after intervention procedure. During the follow-up period, the conditions of all dogs were observed daily. Blood samples were obtained from the right femoral vein at baseline and 3 months after ablation. Three milliliters of blood sample was obtained in an EDTA vial to measure the plasma norepinephrine evaluating the evidence of RDN. After high-speed centrifugation, the blood samples were stored at −80 °C until assay. The plasma norepinephrine was assayed by high-performance liquid chromatography (HPLC).

### Animal Preparation and Renal Angiography

On the day of experiment, the dogs were anesthetized with 3% sodium pentobarbital (30 mg/kg) intraperitoneally, followed by continuously slow injection of sodium pentobarbital through right femoral vein at a maintenance dosage of 5 mg/kg/h with the use of trace syringe pump. Penicillin was given intramuscularly before and after the ablation procedure for preventing infection. After induction of anesthesia, right femoral artery was punctured under sterile conditions, and an 8 F sheath was placed. 2000 ΙU heparin was administered via right femoral artery. Invasive BP was measured at the right femoral artery by connecting to the pressure transducer device and LEAD Electrophysiology Management System (Sichuan Jinjiang Electronic Science and Technology Corporation, China) at baseline, and 30 minutes after the ablation procedure in RDN groups (group A and group B) or the angiography procedure in sham group (group C).

For dogs in sham procedure group, only bilateral renal angiography was performed. For dogs in RDN groups, bilateral renal ablation was performed after renal angiography. However, if renal artery abnormalities were found or diameters were below the minimum acceptable size (<4 mm, using inner diameter of 6 F JR4 Judkins catheter as reference), the animal would be eliminated from the study. 30 minutes after bilateral renal ablation, final renal angiography was performed again to evaluate the changes in diameter and morphology of renal artery, and also for the documentation of vessel patency and intact kidney perfusion. Bilateral renal angiography was performed using a 6 F JR4 Judkins catheter (Cordis Corporation Miami, FL, USA). After the above interventional procedures, sheath was gently removed, and manual compression of the femoral arterial access site was performed to stop bleeding. Within 30 minutes following the achievement of hemostasis, the puncture site was also visually monitored and palpated every 5 minutes to evaluate the risk of complications including bleeding and hematoma. Meanwhile, all dogs were rechecked for the incidence of vascular complications at the puncture site the next day following interventional procedures.

### Renal Denervation Procedure

Referred to the dogs assigned in RDN groups, once the anatomy was deemed acceptable, bilateral RDN was performed. The ablation catheter was positioned into each renal artery via femoral access, and ablation was performed from distal to proximal lumen of the main renal artery trunk by point to point burns both longitudinally and rotationally. There were six to ten ablation points for each side depending on the length of the renal artery. Impedance, power and temperature were monitored during the ablation procedure.

In group A, RDN was performed with a 7 F temperature-controlled catheter (Biosense Webster, Diamond Bar, California). The maximum temperature of catheter was set to 55 °C, RF energy of 10 watts was applied, and duration for each ablation point lasted up to 70 seconds.

In group B, RDN was performed using a 7 F externally saline-irrigated catheter (Thermocool^®^ catheter, Celcius Thermocool, Biosense Webster, Diamond Bar, California). The temperature was set to 45°C, RF energy of 10 watts was delivered, and duration for each ablation point was also 70 seconds. Saline was irrigated manually to decrease the temperature of tissue-electrode interface during RF energy delivery, adjusting speed of irrigation according to the visible temperature (negative feedback adjustment).

### Renal Angiograph Evaluation

Renal angiography was analyzed by three independent investigators blinded to the group setting information, and averages of measurement results by the investigators were calculated. Measurements of renal artery diameter were performed using the 6 F JR4 Judkins catheter with an inner diameter of 2 mm as reference. The middle segment of renal artery, located 1 to 1.5 cm away from the ostium but not the ablation sites, was measured. Compared with the measure of renal artery at baseline, diameter measurements at 30 minutes, 1 month, and 3 months after interventions were performed at the segment with same distance away from the ostium. The mean diameter of renal artery (M-RA) was calculated by the following equation: (left renal artery diameter (L-RA) + right renal artery diameter (R-RA))/2.

### Statistical Analysis

Continuous variables were expressed as mean ± SD. To control the influence of anesthesia and reduce the interference from beat-to-beat variance, data related to BP were obtained by calculating the averages of continuous 10 s beat-to-beat measurements, but not the value of single beat monitoring. The differences of ablation parameters between group A and group B were assessed with the use of two-sample Student’s t-test for continuous variables. Comparisons of the differences in renal artery diameter and BP among group A, group B and group C over three months were performed with the use of two-way mixed ANOVA. The three groups as the between-subjects factor (group) and the repeated measurements during three months as the within-subjects factor (time) were considered. For the comparisons of the differences in the changes of BP and renal artery diameter among groups during time points, two-way mixed ANOVA followed by post hoc analysis with LSD-t test was used. The differences in the changes of BP and renal artery diameter among time points within group were analyzed with the use of repeated measures ANOVA also followed by post hoc analysis with LSD-t test. The comparisons of baseline and reduction of plasma norepinephrine concentrations among groups were performed with the use of one-way ANOVA followed by post-hoc analysis if necessary. Simple associations among BP reduction, renal artery vasodilation, and plasma noradrenaline decrease were assessed with Pearson correlation analysis. A two-sided p < 0.05 was regarded statistically significant. All statistical analyses were performed with SPSS statistical software (version17.0, Chicago, IL, USA).

## Results

Thirty Chinese Kunming dogs were randomly assigned in a 2:2:1 ratio to group A undergoing RDN with TC catheter (n = 12) or group B undergoing RDN with saline-irrigated catheter (n = 12) or group C undergoing a sham procedure (n = 6). However, one dog in group A was excluded because of a dual renal artery while one dog in group B was excluded because of the diameter of renal artery less than 4 mm. Finally, 28 dogs with two anatomically eligible renal vessels were enrolled into this animal study and completed 3-month follow up, with 56 renal arteries evaluated. RDN was successfully performed in all dogs assigned in group A and group B. No renal artery dissection, stenosis, and the other vascular complications, including bleeding and hematoma, occurred post interventional procedures in any enrolled dogs.

### Renal Angiogram and Ablation Parameters

**Figure 1:**
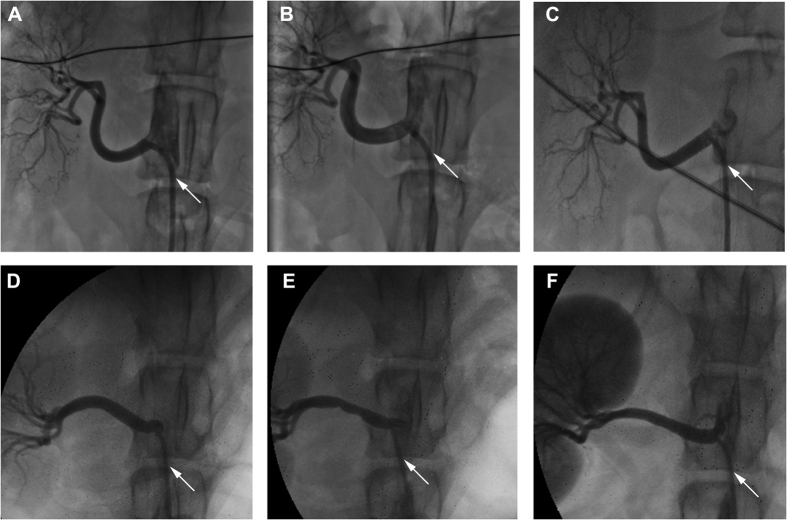
Angiograms before and after ablation. Baseline (**A**) 30-minute (**B**) and 1-month(**C**) renal artery angiograms were from a group B dog. The diameter of renal artery underwent renal nerve ablation via saline-irrigated catheter was markedly increased than that at baseline. Baseline (**D**) 30-minute (**E**) And 1-month (**F**) renal artery angiograms were from a group A dog. Similar changes were showed, but the vessel spasm caused by renal nerve ablation using temperature-controlled catheter made the observation difficult. Angiograms were calibrated using a 6 F JR4 Judkins catheter with an inner diameter of 2 mm as reference (denoted with white arrow heads).

Angiograms revealed the renal artery were smooth in all dogs before ablation. Following RF energy delivery in group A and group B, the renal angiogram showed acute focal irregularities at the ablative site. These ablated lesions were more evident in arteries ablated with TC catheter (group A, Fig. 1), but less apparent in arteries ablated with saline-irrigated catheter (group B, Fig. 1). However, these ablated lesions were transient and disappeared during subsequent follow-up, irrespective of catheter types (Fig. 1).

There were no significant differences in ablation parameters between the RDN groups, viz., average lesion number, average ablation power, and the total radiofrequency delivery time (Table 1). However, the average temperature in group A was much higher than that in group B (49.5 ± 1.5 vs 40.4 ± 1.1 °C, p < 0.001), the average initial impedance in group A was also higher than that in group B (224.6 ± 20.3 vs 204.6 ± 27.2 Ω, p = 0.005), and the decrease of impedance during energy delivery was respectively higher in group A than that in group B (26.2 ± 7.4 vs 20.4 ± 7.5 Ω, p < 0.001) (Table 1).

### Renal Artery Vasodilation

At baseline, the average diameter of right renal artery, left renal artery, and mean diameter were 4.94 ± 0.63 mm, 4.91 ± 0.84 mm, and 4.93 ± 0.63 mm in group A, 4.70 ± 0.64 mm, 4.76 ± 0.63 mm, and 4.73 ± 0.63 mm in group B, and 4.74 ± 0.52 mm, 5.01 ± 0.86 mm, and 4.87 ± 0.66 mm in group C, respectively. There were no significant differences in average diameter of right/left renal artery and mean diameter among groups at baseline with the use of two-way mixed ANOVA followed by post hoc analysis (the main effect of group at baseline: p = 0.632 for right renal artery, p = 0.741 for left renal artery, and p = 0.754 for mean diameter).

Repeated Measures ANOVA test followed by post hoc analyses showed that renal arteries in both group A and group B were significantly dilated during 1–3 months follow up after ablation procedure while the renal arteries in sham group C did not statistically dilate over 3 months follow up (Tables 2 and 3). However, referred to 30 minutes after ablation, the increased renal artery diameter of dogs in group A did not achieve statistical significance comparing with that at baseline, with dilation of 0.138 ± 0.487 mm in right renal artery, 0.214 ± 0.449 mm in left renal artery, and mean dilation of 0.176 ± 0.465 mm (p = 0.368, p = 0.146, p = 0.238 vs. baseline, respectively, Table 3). The diameter of renal artery at 30 minutes post ablation was significantly larger than that at baseline in group B, with dilation of 1.095 ± 0.227 mm in right renal artery, 1.202 ± 0.182 mm in left renal artery, and mean dilation of 1.148 ± 0.190 mm (p < 0.001, p < 0.001, p < 0.001 vs. baseline, respectively, Table 3). After 1 month, in group A, the dilation further increased to 0.872 ± 0.459 mm in right renal artery, 0.984 ± 0.399 mm in left renal artery, and mean dilation of 0.928 ± 0.424 mm (p < 0.001, p < 0.001, p < 0.001 vs. baseline, respectively, Table 3). In group B, the dilation further increased to 1.169 ± 0.221 mm in right renal artery, 1.239 ± 0.170 mm in left renal artery, and mean dilation of 1.204 ± 0.186 mm (p < 0.001, p < 0.001, p <0.001 vs. baseline, respectively, Table 3). After 3 months, vasodilation in group A reached 0.912 ± 0.442 mm in right renal artery, 1.000 ± 0.389 mm in left renal artery, and mean dilation of 0.956 ± 0.410 mm (p < 0.001, p < 0.001, p < 0.001 vs. baseline, respectively, Table 3). In group B, it reached 1.196 ± 0.222 mm in right renal artery, 1.260 ± 0.166 mm in left renal artery, and mean dilation of 1.228 ± 0.186 mm (p < 0.001, p < 0.001, p < 0.001 vs. baseline, respectively, Table 3).

**Figure 2:**
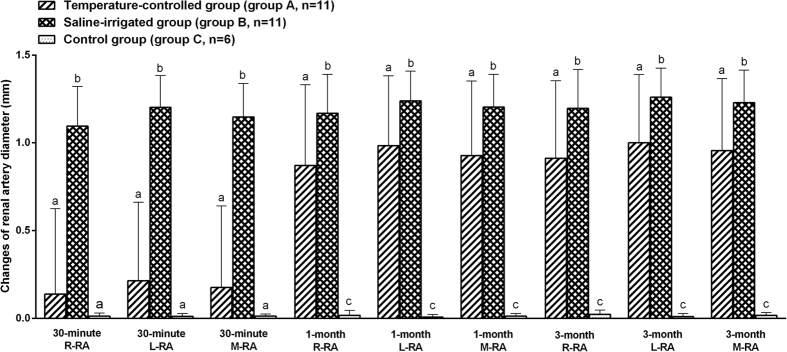
The multiple comparisons of the differences in changes of renal artery diameter among groups during 30 minutes to 3 months follow up after interventions. Renal artery vasodilation was observed in RDN groups (group A and group B), but not in the sham procedure group (group C) during the 30 minutes to 3 months follow up after interventions. Saline-irrigated catheter is superior to TC catheter in renal artery vasodilation, especially for the acute dilatation of renal artery at 30-minute after RDN. The different lowercase letters show significant differences among groups of the variable at 30-minute/1-month/3-month follow up after interventions (LSD post-hoc test at p < 0.05), while the same lowercase letter show no significant differences among groups of the variable (LSD post-hoc test at p > 0.05). R-RA =  diameter of right renal artery; L-RA =  diameter of left renal artery; M-RA = mean diameter of renal artery.

The results of two-way mixed ANOVA followed by post hoc analysis with LSD-t test mainly showed the differences in extent of renal artery dilatation among groups during follow-up time points. At 30 minutes after ablation, the dilatation of renal artery in dogs assigned to saline-irrigated catheter (group B) was much more obvious than that in TC catheter (group A) (p < 0.001 for right renal artery, p < 0.001 for left renal artery, and p < 0.001 for mean diameter) and sham procedure (group C) (p < 0.001 for right renal artery, p < 0.001 for left renal artery, and p < 0.001 for mean diameter), while there were no significant differences between TC catheter group and sham procedure group (p = 0.476 for right renal artery, p = 0.207 for left renal artery, and p = 0.320 for mean diameter) (Fig. 2). At 1 month after ablation, the dilatation of renal artery in TC catheter group was statistically more obvious than that in sham procedure group (p < 0.001 for right renal artery, p < 0.001 for left renal artery, and p < 0.001 for mean diameter), but less than that in saline-irrigated catheter group (p = 0.040 for right renal artery, p = 0.039 for left renal artery, and p = 0.036 for mean diameter) (Fig. 2). At 3 months after ablation, saline-irrigated catheter (group B) was still superior to TC catheter (group A) in extend of renal artery vasodilation (p = 0.043 for right renal artery, p = 0.031 for left renal artery, and p = 0.034 for mean diameter), while the dilatation of renal artery in TC catheter group was also statistically more obvious than that in sham procedure group (p < 0.001 for right renal artery, p < 0.001 for left renal artery, and p < 0.001 for mean diameter) (Fig. 2).

### Reduction of Blood Pressure

At baseline, systolic/diastolic BP (SBP/DBP) was 166 ± 19/101 ± 10 mmHg in group A, 169 ± 16/105 ± 12 mmHg in group B, and 178 ± 18/110 ± 7 mmHg in group C. There were no significant differences in SBP/DBP among groups at baseline with the use of two-way mixed ANOVA followed by post hoc analysis (the main effect of group at baseline: p = 0.450 for SBP, p = 0.330 for DBP).

**Figure 3:**
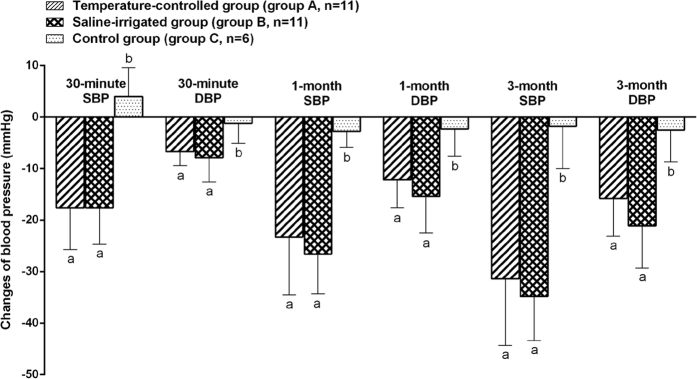
The multiple comparisons of the differences in changes of blood pressure among groups during 30 minutes to 3 months follow up after interventions. Systolic/diastolic blood pressure in RDN groups (group A and group B) was significantly decreased after RDN while no significant changes were observed in sham procedure group (group C). The different lowercase letters show significant differences among groups of the variable at 30-minute/1-month/3-month follow up after interventions (LSD post-hoc test at p < 0.05), while the same lowercase letter show no significant differences among groups of the variable (LSD post-hoc test at p > 0.05). SBP = systolic blood pressure; DBP = diastolic blood pressure.

Repeated Measures ANOVA test showed that BP in RDN groups (group A and group B) was significantly decreased after RDN while no significant changes were observed in sham procedure group (group C) (Table 2). BP (SBP/DBP) reduction in group A were from −17.6 ± 8.1/−6.2 ± 2.7 mmHg to −23.3 ± 11.2/−12.2 ± 5.4 mmHg, and −31.4 ± 12.9/−15.8 ± 7.3 mmHg at 30-minutes, 1-month, and 3-month (p < 0.001, p < 0.001, p < 0.001 vs. baseline for SBP and DBP, respectively; Table 3). Likewise, as shown in Table 3, BP in group B decreased by an average of −17.6 ± 7.1, −26.6 ± 7.7, and −34.8 ± 8.6 mmHg for SBP (p < 0.001, p < 0.001, p < 0.001 vs. baseline, respectively), −8.0 ± 4.7, −15.4 ± 7.1, and −21.1 ± 8.2 mmHg for DBP (p < 0.001, p < 0.001, p < 0.001 vs. baseline, respectively). Two-way mixed ANOVA followed by post hoc analysis showed that the BP reduction in RDN groups (group A and group B) was statistically more obvious than that in sham procedure group (group C) during follow-up time points, however, no significant differences were observed between group A and group B (at 30-minute: p = 0.981 for SBP, p = 0.434 for DBP; at 1-month: p = 0.389 for SBP, p = 0.235 for DBP; at 3-month: p = 0.455 for SBP, p = 0.109 for DBP) (Fig. 3).

### The Level of Plasma Norepinephrine

The plasma norepinephrine concentrations at baseline were 1.30 ± 0.32 nmol/L in group A, 1.48 ± 0.25 nmol/L in group B, and 1.59 ± 0.36 nmol/L in group C (p = 0.142 for the main effect of group at baseline). The plasma norepinephrine concentrations significantly decreased to 0.56 ± 0.11 nmol/L in group A and 0.55 ± 0.13 nmol/L in group B (p = 0.933 for group A vs group B), but slightly increased to 1.67 ± 0.38 nmol/L in group C at 3-month follow up after randomization (p < 0.001 for group C vs group A, p < 0.001 for group C vs group B). The reduction of plasma norepinephrine during 3 months after ablation between group A and group B didn’t show significant difference (53.3 ± 16.7 vs 60.6 ± 15.9%, p = 0.623).

### Correlation among BP Reduction, Renal Artery Vasodilation, and Plasma Norepinephrine Reduction 3 Months after RDN

RDN groups showed significant BP reduction, renal artery vasodilation, and plasma norepinephrine reduction 3 months after ablation. Correlation analyses showed that there were significant positive correlations between SBP reduction and renal artery vasodilation (r = 0.69 and p < 0.001 for ΔSBP and ΔR-RA, r = 0.74 and p < 0.001 for ΔSBP and ΔL-RA, r = 0.72 and p < 0.001 for ΔSBP and ΔM-RA), renal artery vasodilation and plasma norepinephrine reduction (r = 0.66 and p < 0.01 for Δnorepinephrine and ΔR-RA, r = 0.70 and p < 0.001 for Δnorepinephrine and ΔL-RA, r = 0.69 and p < 0.001 for Δnorepinephrine and ΔM-RA), and SBP reduction and plasma norepinephrine reduction over 3 months after RDN (r = 0.95 and p < 0.001 for ΔSBP and Δnorepinephrine) (Table 4).

## Discussion

The question addressed by present study was that once renal sympathetic fibers were damaged or sufficiently injured by the TC catheter or saline-irrigated catheter, in addition to BP reduction, whether the renal artery would be dilated. The main findings of this study are that after the interruption of renal sympathetic nervous system by RF ablation regardless of catheter types, besides the effectiveness of BP reduction, renal artery vasodilation can be observed by angiography. Furthermore, there are significant positive correlations between SBP reduction and renal artery vasodilation, renal artery vasodilation and plasma norepinephrine reduction, and SBP reduction and plasma norepinephrine reduction at 3-month after RDN. In addition, saline-irrigated catheter is superior to TC catheter in renal artery vasodilation, especially for the acute dilatation of renal artery at 30-minute after RDN.

The main findings of this study re-illustrate that RDN can achieve significant reduction of BP. Previous morphologic studies showed that all essential renal structures, including the renal vasculature, the tubules, and the juxta-glomerular apparatus, are dominantly innervated by renal sympathetic nerve fibers^20^,^21^,^23^. Renal sympathetic activation leads to volume retention via increased sodium reabsorption, reduction of renal blood flow, and activation of the renin-angiotensin-aldosterone system via increased renin release from the juxta-glomerular apparatus, which are important for BP regulation^29^,^30^,^31^. Theoretically speaking, sufficient disruption of sympathetic fibers via RDN is an effective non-pharmacological method to lower BP, which has been revealed by previous clinical researches^7^,^8^,^10^,^11^. Meanwhile, our previous published basic study had shown that catheter-based RDN, no matter conducted by TC catheter or saline-irrigated catheter, could induce the significant damage to renal nerves^28^. The manifestations of the damages induced by radiofrequency energy to renal nerves included the denaturation, demyelination of neural fibers and axons evaluated by histopathological analysis, and also the vacuolization, electron-dense deposits, hyperplasia and hypertrophy of Schwann cells of neural fibers and axons evaluated by the transmission electron microscopy^28^. Additionally, the damage of renal nerves produced by catheter-based RDN had also been demonstrated by Wang *et al.*^32^ and Li *et al.*^33^ using the immunohistochemical staining of sympathetic nerves. Therefore, above evidences from animal studies and clinical researches indicated that catheter-based RDN should have good application prospects as a potential device-based therapeutic option for patients with hypertension by targeting the renal nerves to reduce BP.

As previously assumed, RDN induced significant renal artery vasodilation in our study. Previous studies showed that the vasoconstriction/vasodilation status of the renal artery was modulated by renal sympathetic nerve^17^,^19^,^22^,^34^. After RDN, other than marked and sustained BP reduction, a decrease in renin activity and an increase in renal plasma flow have already been reported^35^,^36^. Mahfoud^37^ and Tsioufis^36^ both showed that RDN reduced renal resistive index, which was potentially related to decreased renal sympathetic tone, inducing vasodilation. Consistent with our findings, renal angiograms in Rippy’s study^38^ also showed renal artery vasodilation during the further follow-up period. In our study, we further performed the correlation analysis between renal artery vasodilation and plasma norepinephrine reduction. The results showed that there was a significant positive correlation between renal artery vasodilation and plasma norepinephrine reduction which is considered as the direct evidence of sympathetic nerve injury. These results imply that RDN induces significant renal artery vasodilation by interrupting the renal sympathetic nerves.

Contrary to our findings, morphological study by Steigerwald *et al.*^39^ and OCT imaging by Templin *et al.*^24^ both showed that circumscribed vascular spasm and local tissue damage at the ablation site with edema and thrombus formation after RDN could lead to a significant reduction in the mean renal artery diameter. In regards to this discrepancy, we reckoned that the effect of RDN on renal artery may be the same among these studies, with the only differences in its extent and catheter used. Theoretically speaking, after sufficient disturbance of the renal nerves, instantaneous vasodilation should be observed. However, due to the acute local vascular injury induced by the RF energy, few studies have documented vasodilation, especially at acute phase. Likewise in our study, irrespective of catheter types, RDN decreased BP significantly in both groups. However, the transient ablated lesions, which disappeared during subsequent follow-ups, were more evident in arteries ablated with TC catheter. As our published data shown, compared with saline-irrigated catheter, TC catheter produces much more significant injury and hyperplasia to the renal artery intima with much less and shallower destruction to renal sympathetic nerve^28^. Thus, renal artery vasodilation induced by TC catheter, potentially related to the destruction of sympathetic nerve produced by RDN, could be counteracted by acute local vascular injury.

Additionally, plasma norepinephrine concentrations were significantly decreased in RDN groups, no matter conducted by TC catheter or saline-irrigated catheter. Besides shown in present study, previous published researches also showed that catheter-based RDN could not only decrease the renal norepinephrine level but also simultaneously reduce the plasma norepinephrine concentrations^35^. It has been demonstrated that renal nerves consist of renal efferent sympathetic nerves and also the renal sensory afferent nerves^40^. Meanwhile, the research conducted by Bleys RL *et al.* showed that renal efferent sympathetic nerves and renal sensory afferent nerves were mostly contained into the same nerve bundle traveling around in renal artery adventitia^40^. Therefore, catheter-based RDN is proposed to destroy both renal efferent sympathetic nerves and also the renal sensory afferent nerves by radiofrequency energy delivery. The activation of renal sensory afferent nerve is thought to have central effects and involve in central mechanisms regulating the sympathetic outflow to the heart and the peripheral circulation^5^,^41^,^42^. Thus, referred to the reasons of RDN reducing the circulation norepinephrine level, theoretically, it is probably because that catheter-based RDN, through damaging the renal afferent nerves, influences the central regulatory mechanisms of autonomic nervous system and decreases the sympathetic outflow to the peripheral circulation system^41^,^42^. Similar with the effect of RDN on circulation norepinephrine levels, there is evidence indicating that RDN can also decrease the muscle sympathetic nerve activity^43^ as well as cardiac sympathetic activity^44^. In other words, RDN can also effectively decrease the extra-renal sympathetic nerve activity by damaging renal afferent nerve and correspondingly decreasing the sympathetic outflow from brain to the peripheral circulation system.

Based on the above discussions, RDN technique is supported by affluent theoretical foundation. However, it is known that SYMPLICITY HTN-3, a single-blind, randomized, sham-controlled clinical trial aimed to investigate the efficacy and safety of RDN on hypertension, failed to meet its primary efficacy endpoint^12^. After in-depth analysis of SYMPLICITY HTN-3 data and careful summarizing of the RDN experience, some problems requiring urgent solution in reference to RDN technique were proposed by worldwide experts. Among these problems, lack of indicator or predictor of successful renal sympathetic nerve damage on spot was one of the problems in focus^45^,^46^. In this study, the vasodilation of renal artery was observed after RDN, especially for the saline-irrigated catheter group, and the advanced correlation analyses results showed that renal artery vasodilation positively correlated with SBP reduction and plasma norepinephrine reduction over 3 months after ablation. These findings imply that renal artery vasodilation may be the indicator of successful renal sympathetic nerve damage during RDN procedure and a predictor of RDN related to BP response post procedure.

As a basic research related to catheter-based RDN targeting sympathetic nerves to reduce BP, the selection of experimental animals with natural hypertension and excessive sympathetic activity is very crucial to test our hypotheses. To perform the percutaneous catheter-based RDN technique, large experimental animal model was required. As previously described, the Chinese Kunming dogs are larger, more aggressive and fierce than beagles, spaniels and mongrels, more importantly having high natural BP and sympathetic activity^25^,^26^. Thus, this study was performed on Chinese Kunming dogs. Using the Chinese Kunming dogs as the experimental animals, we had successfully performed a series of basic researches related to catheter-based RDN technique^25^,^27^,^28^. During the previous peer review procedures of the above published animal studies, the use of Chinese Kunming dogs with “spontaneous” hypertension and natural excessive sympathetic activity had been proposed as a highlights of our researches by several reviewers. Similar with Chinese Kunming dogs, the Guizhou mini-pig was also proposed as a novel spontaneous hypertensive animal model with sympathetic hyperactivity and responded well to catheter-based RDN^33^. Therefore, using these large experimental animals with spontaneous hypertension and excessive sympathetic activity, we hope that the recently proposed urgent problems of catheter-based RDN technique, such as the indicator or predictor of successful RDN, endpoints of RDN procedure, and the optimal indication of RDN, can be intensively investigated and systematically answered in the near future.

### Study Limitations

Some limitations should be considered in interpreting the present results. A major limitation of this study is the small sample size, and although vascular diameter measurement is conducted by independent investigators blinded to the experiment, owing to inter observer variability, a larger sample size of the experiment may be needed. Meanwhile, the invasive blood pressure measurements under anesthesia and no direct histopathological evidence referred to the renal sympathetic nerve damage should also be listed as limitations of this study. However, the direct evidence of renal nerve damages induced by catheter-based RDN, had been shown in previous published researches^28^,^32^,^33^. Additionally, because this is an animal study, the analyses results from the on-going SWAN-HT Study (identifier: NCT01417221) are awaited with great interest to confirm the findings of the present study.

## Conclusions

In conclusion, besides the effectiveness of BP reduction, RDN also induces significant renal artery vasodilation by damaging renal sympathetic nerve. The renal artery vasodilation, as a possible indicator of successful renal sympathetic nerve damage during the RDN procedure and a predictor of efficient BP response, positively correlates with SBP reduction and plasma norepinephrine reduction over 3 months after ablation.

## Additional Information

**How to cite this article**: Chen, W. *et al.* Renal Artery Vasodilation May Be An Indicator of Successful Sympathetic Nerve Damage During Renal Denervation Procedure. *Sci. Rep.* **6**, 37218; doi: 10.1038/srep37218 (2016).

**Publisher’s note:** Springer Nature remains neutral with regard to jurisdictional claims in published maps and institutional affiliations.

## Figures and Tables

**Table 1:** Comparisons of ablation parameters between two RDN groups.

Procedure Variables	Temperature-controlled group (group A, n = 11)	Saline-irrigated group (group B, n = 11)	P value
Lesion NO.	14.8 ± 1.3	15.0 ± 1.1	0.73
Total Radiofrequency(S)	1037.3 ± 92.9	1050 ± 76.7	0.73
Average Power(W)	9.9 ± 0.03	9.9 ± 0.09	0.534
Average Temperature(°C)	49.5 ± 1.5	40.4 ± 1.1	<0.001
Average Initating Impedance(Ω)	224.6 ± 20.3	204.6 ± 27.2	0.005
Δ Impedance(Ω)	26.2 ± 7.4	20.4 ± 7.5	<0.001

Values were presented as mean ± SD. The comparisons of ablation parameters between two groups were performed with the use of two-sample Student’s t-test for continuous variables. Δ Impedance means the average decrease value of impedance during energy delivery.

**Table 2:** Blood pressure and diameter of renal artery at baseline, 30 minutes, 1 month and 3 months.

	Temperature-controlled group (group A, n = 11)					Saline-irrigated group (group B, n = 11)					Control group (group C, n = 6)				
	Baseline	30 minutes	1 Month	3 months	p value	Baseline	30 minutes	1 Month	3 months	p value	Baseline	30 minutes	1 Month	3 months	p value
Blood pressure															
SBP (mmHg)	166 ± 19	149 ± 15	143 ± 13	135 ± 11	<0.001	169 ± 16	152 ± 12	143 ± 15	135 ± 13	<0.001	178 ± 18	182 ± 21	175 ± 17	176 ± 18	0.180
DBP (mmHg)	101 ± 10	95 ± 9	89 ± 8	86 ± 6	<0.001	105 ± 12	97 ± 11	89 ± 10	84 ± 10	<0.001	110 ± 7	108 ± 5	107 ± 6	107 ± 2	0.615
Renal artery diameters															
R-RA (mm)	4.94 ± 0.63	5.08 ± 0.95	5.81 ± 0.95	5.85 ± 0.94	<0.001	4.70 ± 0.64	5.79 ± 0.74	5.87 ± 0.80	5.89 ± 0.79	<0.001	4.74 ± 0.52	4.75 ± 0.52	4.76 ± 0.53	4.76 ± 0.51	0.215
L-RA (mm)	4.91 ± 0.84	5.13 ± 0.94	5.90 ± 0.91	5.91 ± 0.90	<0.001	4.76 ± 0.63	5.96 ± 0.72	5.99 ± 0.71	6.02 ± 0.70	<0.001	5.01 ± 0.86	5.02 ± 0.85	5.01 ± 0.85	5.02 ± 0.84	0.513
M-RA (mm)	4.93 ± 0.63	5.10 ± 0.94	5.85 ± 0.93	5.88 ± 0.92	<0.001	4.73 ± 0.63	5.88 ± 0.73	5.93 ± 0.75	5.96 ± 0.74	<0.001	4.87 ± 0.66	4.89 ± 0.66	4.89 ± 0.67	4.89 ± 0.65	0.176

Values were presented as mean ± SD. Comparisons of the differences in renal artery diameter and BP over 3 months within group were performed with the use of repeated measures ANOVA test. SBP =  systolic blood pressure; DBP =  diastolic blood pressure; R-RA =  diameter of right renal artery; L-RA =  diameter of left renal artery; M-RA = mean diameter of renal artery.

**Table 3:** Changes of blood pressure and renal artery diameter from baseline to 30 minutes, 1 month, and 3 months.

	Temp-controlled group (group A, n = 11)			Saline-irrigated group (group B, n = 11)			Control group (group C, n = 6)		
	30 minutes	1 Month	3 months	30 minutes	1 Month	3 months	30 minutes	1 Month	3 months
Changes of blood pressure									
Δ SBP (mmHg)	17.6 ± 8.1*	23.3 ± 11.2*	31.4 ± 12.9*	17.6 ± 7.1*	26.6 ± 7.7*	34.8 ± 8.6*	4.0 ± 5.6†	2.8 ± 3.1†	1.8 ± 8.2†
Δ DBP (mmHg)	6.7 ± 2.7*	12.2 ± 5.4*	15.8 ± 7.3*	8.0 ± 4.7*	15.4 ± 7.1*	21.1 ± 8.2*	1.2 ± 3.9†	2.3 ± 5.3†	2.5 ± 6.2†
Changes of renal artery diameters									
Δ R-RA (m m)	0.138 ± 0.487†	0.872 ± 0.459*	0.912 ± 0.442*	1.095 ± 0.227*	1.169 ± 0.221*	1.196 ± 0.222*	0.013 ± 0.018†	0.018 ± 0.029†	0.023 ± 0.024†
Δ L-RA (mm)	0.214 ± 0.449†	0.984 ± 0.399*	1.000 ± 0.389*	1.202 ± 0.182*	1.239 ± 0.170*	1.260 ± 0.166*	0.012 ± 0.016†	0.007 ± 0.016†	0.010 ± 0.018†
Δ M-RA (mm)	0.176 ± 0.465†	0.928 ± 0.424*	0.956 ± 0.410*	1.148 ± 0.190*	1.204 ± 0.186*	1.228 ± 0.186*	0.013 ± 0.012†	0.013 ± 0.015†	0.017 ± 0.017†

Values were presented as mean ± SD. Comparisons of the changes in BP and renal artery diameter between the 30-minute/1-month/3-month follow up and baseline were performed with the use of repeated measures ANOVA followed by LSD-t test.^*^means p < 0.05 comparing with that at baseline; ^†^means p > 0.05 comparing with that at baseline. ΔSBP = change of systolic blood pressure; ΔDBP = change of diastolic blood pressure; ΔR-RA = change of right renal artery diameter; ΔL-RA = change of left renal artery diameter; ΔM-RA = change of mean diameter of renal artery.

**Table 4:** Correlation among blood pressure reduction, renal artery vasodilation, and plasma norepinephrine reduction over 3 months after RDN.

	Δ SBP	Δ DBP	Δ M-RA	Δ NE	Δ R-RA	Δ L-RA
Δ SBP	1.00	0.45*	0.72***	0.95***	0.69***	0.74***
Δ DBP	0.45*	1.00	0.47*	0.53*	0.46*	0.46*
Δ M-RA	0.72***	0.47*	1.00	0.69***	0.99***	0.98***
Δ NE	0.95***	0.53*	0.69***	1.00	0.66**	0.70***
Δ R-RA	0.69***	0.46*	0.99***	0.66**	1.00	0.94***
Δ L-RA	0.74***	0.46*	0.98***	0.70***	0.94***	1.00

Simple associations among BP reduction, renal artery vasodilation, and plasma noradrenaline decrease were assessed with Pearson correlation analysis. RDN = renal denervation; ΔSBP = change of systolic blood pressure; ΔDBP = change of diastolic blood pressure; ΔR-RA = change of right renal artery diameter; ΔL-RA = change of left renal artery diameter; ΔM-RA = change of mean diameter of renal artery; ΔNE =  change of plasma norepinepherine. Note. ^*^equal p < 0.05; ^**^equal p < 0.01; ^***^equal p < 0.001.
